# *Eriobotrya japonica *hydrophilic extract modulates cytokines in normal tissues, in the tumor of Meth-A-fibrosarcoma bearing mice, and enhances their survival time

**DOI:** 10.1186/1472-6882-11-9

**Published:** 2011-02-04

**Authors:** Heba A Alshaker, Nidal A Qinna, Fadi Qadan, Mona Bustami, Khalid Z Matalka

**Affiliations:** 1 Department of Pharmacology and Biomedical Sciences, Faculty of Pharmacy and Medical Sciences, Petra University, Amman, Jordan; 2 Department of Pharmaceutical Medicinal Chemistry and Pharmacognosy, Faculty of Pharmacy, Petra University, Amman, Jordan

## Abstract

**Background:**

Cytokines play a key role in the immune response to developing tumors, and therefore modulating their levels and actions provides innovative strategies for enhancing the activity of antigen presenting cells and polarizing towards T helper 1 type response within tumor microenvironment. One of these approaches could be the employment of plant extracts that have cytokine immunomodulation capabilities. Previously, we have shown that the *Eriobotrya japonica *hydrophilic extract (EJHE) induces proinflammatory cytokines *in vitro *and *in vivo*.

**Methods:**

The present study explored the *in vivo *immunomodulatory effect on interferon-gamma (IFN-γ), interleukin-17 (IL-17), and transforming growth factor-beta 1 (TGF-β1) evoked by two water-extracts prepared from EJ leaves in the tissues of normal and Meth-A-fibrosarcoma bearing mice.

**Results:**

Intraperitoneal (i.p.) administration of 10 μg of EJHE and EJHE-water residue (WR), prepared from butanol extraction, increased significantly IFN-γ production in the spleen (p < 0.01) and lung (p < 0.03) tissues at 6-48 hours and suppressed significantly TGF-β1 production levels (p < 0.001) in the spleen for as long as 48 hours. The latter responses, however, were not seen in Meth-A fibrosarcoma-bearing mice. On the contrary, triple i.p. injections, 24 hours apart; of 10 μg EJHE increased significantly IFN-γ production in the spleen (p < 0.02) while only EJHE-WR increased significantly IFN-γ, TGF-β1 and IL-17 (p < 0.03 - 0.005) production within the tumor microenvironment of Meth-A fibrosarcoma. In addition, the present work revealed a significant prolongation of survival time (median survival time 72 days vs. 27 days of control, p < 0.007) of mice inoculated i.p. with Meth-A cells followed by three times/week for eight weeks of i.p. administration of EJHE-WR. The latter prolonged survival effect was not seen with EJHE.

**Conclusions:**

The therapeutic value of EJHE-WR as an anticancer agent merits further investigation of understanding the effect of immunomodulators' constituents on the cellular components of the tissue microenvironment. This can lead to the development of improved strategies for cancer treatment and thus opening up a new frontier for future studies.

## Background

Multiple innate and adaptive immune effector cells and molecules partake in the recognition and destruction of cancer cells to protect against growing tumors, a concept that is known as cancer immune surveillance. Unfortunately, cancer cells are capable of avoiding this process by immunoselection of poorly immunogenic tumor cells along with subversion of the immune system and thus shaping both the tumor and its microenvironment [1]. Cytokines represent part of the complex pattern of the immune response which can assist the development of cancer as well as to eliminate it. Simultaneously, a large number of cytokines may be involved in the complex interactions between host and tumor cells where this dynamic crosstalk, between tumors and the immune system, can regulate tumor growth and metastasis [2].

The failure of the immune system to recognize and eradicate cancer cells may partly be a result of insufficient immunological activation. It is now increasingly recognized that the microenvironment plays a critical role in the progression of tumors where immune-resistant tumor variants are selected, thereby initiating the process of cancer immunoediting. One variable that might prove conclusive in modulating the host reaction is the mixture of cytokines produced within tumor microenvironment [2,3]. Meanwhile, local activation of the immune system at the tumor microenvironment site can be achieved by experimental transfer and expression of cytokine genes in tumor cells [2,3]. Interestingly, cytokines in cancer can function in bidirectional way as both; causative agents and potential treatments thusly necessitating in-depth knowledge of the role of all the various cytokines which in turn provides new opportunities for improving cancer immunotherapy.

Several medicinal plants are considered immunomodulatory as they display cytokine modulation and have antitumoral effects. Of these are the leaves of *Eriobotrya japonica *EJ Lindl (Rosaceae). Traditionally, EJ has been used for treating lung, stomach diseases and diabetes and has been found to be effective in chronic bronchitis, inflammatory diseases, and against certain tumors [4-8]. A number of biologically active compounds have been reported in the leaves of this plant for instance, polyphenols, tannins, flavonoids, triterpenes, megastigmane glycosides, and sesquiterpenes [4-6,9]. Many of these biologically active compounds were correlated to cytotoxic, antimutagenic and antitumor effects in mouse tumor models and human oral tumor cell lines [4-6].

Previously, we have shown that the hydrophilic leaf extract of EJ, EJHE, induces and modulates *in vitro *and *in vivo *proinflammatory cytokines production, IL-12, IFN-γ and tumor necrosis factor-alpha (TNF-α) more than an anti-inflammatory cytokine, IL-10 [10]. The present study extended the latter observation of EJHE modulatory effects on IFN-γ, TGF-β1 as a representative of T regulatory (Treg) cells, and IL-17 as an indicator of T helper (Th) 17 cells in normal tissues as well as in Meth-A-fibrosarcoma tumor-bearing mice [11]. It has been shown that IFN-γ, IL-17 and TGF-β1 are capable of exerting multiple effects within the tumor microenvironment [12-14]. Controversially, many of the exerted effects are contextual hence, compelling further investigations in that regard along with any attempt of their immunomodulation that should be approached with a definite knowledge of the initial level of cytokines and their complex interaction with particular type of tumors and the immune cells.

Taken into account the extraction method which provokes key role in natural products and ultimately may or may not modulate the immune function EJHE was further extracted with n-butanol. The non-extracted material was referred to as water residue (EJHE-WR). EJHE-WR has been shown to induce more of IFN-γ than EJHE [10]. EJHE or EJHE-WR provides possible ways to reestablish antitumor immune response which can be further exploited for cancer therapy and thereby reducing the complications due to toxicity associated with cytokines systemic administration [15]. Altogether, the main objective of this study was to examine EJHE and EJHE-WR possible treatment of Meth-A, 3-methylcholanthrene (MCA)-induced fibrosarcoma, through the manipulation of the cytokine triad mentioned previously along with deeper exploration of this cytokine trilogy synergistic or antagonistic interactions with respect to tumor immunity at the tumor microenvironment.

## Methods

### Plant material

Fresh leaves of EJ LINDL (Rosaceae) were collected from Jabal Al Hussein area in Amman-Jordan (06/2009), washed extensively with tap water, dried for one week at room temperature, and then grounded into powder. The plant material was identified in comparison with authentic EJ obtained from the Botanical Institute, University of Cologne (Germany).

### Plant material extraction

Five hundred grams of powdered leaves were thoroughly extracted with boiled water. In portions, boiled water (300 ml) was added to 50 g powder for five minutes followed by filtration into a flask. This step was repeated three times. The native liquid EJHE was combined at the end of this step giving a total volume of 2 liters. A part of the combined native liquid EJHE (300 ml) was freeze dried to obtain 8.93 g of the EJHE. The remaining (1700 ml) of the combined native liquid EJHE was successively partitioned with n-butanol (1:1). Each portion of the remaining native EJHE was repeatedly extracted (three times or until solution became clear) with n-butanol. The water phase, non-extracted material, was combined at the end of this step and referred to as EJHE-WR. A part of the combined liquid EJHE-WR (300 ml) was freeze dried to obtain 10.76 g of the EJHE-WR.

Before utilization in animal testing, each of EJHE or EJHE-WR was dissolved in endotoxin-free phosphate buffered saline (PBS) and sterilized through filtration with sterile filters (0.2 μm). Then, the required concentrations were subsequently prepared in endotoxin-free PBS and used fresh for each experiment.

### Materials for cytokines extraction, assays and establishment of tumor cell line

Endotoxin-free Dulbecco's PBS without calcium and magnesium, PRMI-1640, fetal bovine serum (FBS), and penicillin-streptomycin were purchased from Euroclone (Siziano, Italy). Trypsin was obtained from PAA Laboratories Gmbh (Linz, Austria). Igepal CA-630, MCA 98%, and lipopolycaccharide (LPS, L-6143) were obtained from Sigma (St. Louis, MO, USA). T75 flasks were purchased from Costar (Cambridge, MA, USA). Maxisorb 96-well flat bottom plates were purchased from Nunc International (Roskilde, Denmark).

### Mice

BALB/c males and females mice were utilized throughout the experiments as indicated. BALB/c mice were purchased from Yarmouk University (Irbid, Jordan). Animals were housed on a 12:12 hr light cycle at 22 ± 2°C with food and water available *ad libitum*. All animal experiments were performed in compliance with relevant laws and institutional guidelines following the approval of the Ethics Committee of Petra University (Amman, Jordan).

### Cytokine extraction following administration of extracts

To choose the best possible dose for the cytokine-induced modulation over time by the two extracts, four different doses/5 mice/group prepared in 1 ml of sterile PBS were chosen 0, 1, 10 and 100 μg of EJHE and EJHE-WR and were administered i.p. into 8-10 weeks old mice. After 2 hours, mice were sacrificed by cervical dislocation and tissues/organs were collected within 5-10 minutes. The collected tissues/organs were blood, lungs, and spleen. Post sacrificing each mouse blood was directly collected from the cardiac chamber, placed into pre-chilled tube, weighed and incubated with 2 ml of ice-cold endotoxin-free PBS containing 0.1% Igepal CA-630 under ice [11]. After blood collection, the lungs, spleen, and tumor were removed from mice, weighed, placed in pre-chilled tube containing 2 ml of ice-cold endotoxin-free PBS with 0.1% Igepal CA-630, and incubated for 10 minutes on ice. The tissues were then homogenized with a tissue disrupter (Janke and Kundle), centrifuged (6000 rpm for 6 minutes), and the supernatant was transferred to labeled microcentrifuge tubes and stored -30°C till cytokine assays [11].

The modulatory effect of 10 μg EJHE or EJHE-WR on IFN-γ, IL-17 and TGF-β1 tissue levels and in comparison to 2 μg of LPS were performed using fifty-two male mice of 8-10 weeks old. Following a single i.p. injection, mice were sacrificed from each group by cervical dislocation at specific time points (0, 2, 6, 24 and 48 hours) and blood, lungs, and spleen were collected as described above.

### MCA-induced tumors, cell lines preparation and inoculation

Mice (2-4 weeks old) were inoculated subcutaneously (s.c.) into the right hind flank with 1 mg/mouse of MCA dissolved in olive oil. Mice were inspected weekly for tumor development. When tumors reached a 1-2 cm in diameter, mice were sacrificed and the primary MCA-induced tumors were aseptically excised, cut into small pieces, minced, treated with trypsin, and cultured in T75 flasks containing PRMI-1640 medium supplemented with 10% FBS, and antibiotics (100 U/ml penicillin and 100 μg/ml streptomycin), at 37°C in a humidified atmosphere of 5% CO_2_. The medium was changed every 2-3 days. Cells were split by trypsinization to maintain logarithmic growth.

For inoculation, the harvested Meth-A cells were counted and resuspended in endotoxin-free PBS to the desired concentrations. A suspension of Meth-A cells was inoculated s.c. in the right flank of the mouse to establish a tumor model of Meth-A fibrosarcoma. In this experimental system, twenty-one male mice and thirty female mice were inoculated with 2 × 10^6 ^tumor cells in 200 μl endotoxin-free PBS. Recipient mice were inspected twice weekly. Tumor-bearing mice were defined by the presence of a progressively growing tumor with a diameter of ≥7 mm measured using a digital caliper. Noteworthy, following tumor inoculation the mice behaved normally, no apparent suffering, and no signs of pain or other disorders were noticed.

### Cytokine modulation in Meth-A-tumor-bearing mice after administration of EJHE and EJHE-WR

Tumor-bearing mice with progressively growing transplanted-tumors were divided into five groups each consisting of three to five mice. Groups 1 and 2 were administered i.p. with 1 ml of 10 μg EJHE and EJHE-WR, respectively. Group 3 was administered i.p. with 1 ml of endotoxin-free PBS and considered as the control group. Following 24 hours, mice from groups 1, 2, and 3 were sacrificed. Groups 4 and 5 were administered i.p. with 1 ml of 10 μg EJHE and EJHE-WR, respectively, once every 24 hours for three consecutive days and then sacrificed on the fourth day. Mice were sacrificed by cervical dislocation then spleen and tumor were collected for cytokines extraction as described above. This experiment was performed twice and the pattern of cytokine change was the same in the two experiments. Therefore, the data presented here were derived from a single experiment

### Survival of Meth-A tumor-bearing mice after administration of EJHE and EJHE-WR

Thirty female mice were divided into three groups each consisting of ten mice. Mice were inoculated i.p. with 2 × 10^6 ^Meth-A cells in 0.5 ml endotoxin-free PBS. Three days after tumor inoculation, group 1 and 2 were administered i.p. with 0.5 ml of 10 μg EJHE and EJHE-WR, respectively. Group 3 was administered i.p. with 0.5 ml of endotoxin-free PBS and considered as the control group. The mice in the three groups were repeatedly administered for eight weeks with the corresponding doses every 48 hours with one day off following each three consecutive administrations. The weights of the mice were documented before each administration. The mice in each group were observed daily to monitor their survival. The survival time of the mice was also recorded.

### Cytokine assays

Measurements of mouse tissue-extracted cytokines (IFN-γ, IL-17, and TGF-β1) were accomplished by sandwich ELISAs developed in accordance with the manufacturer's recommendations (Duoset R & D Systems, UK. Plates were read at 450 nm by SCO GmbH (Dingelsadt, Germany) ELISA plate reader and absorbance was transformed to cytokines concentrations (pg/ml) and then to (pg/g) of tissues using a standard curve computed on Excel sheet after transforming values to log to construct a straight line on a log-log graph.

### Statistical analysis

All data in the figures are presented as the mean ± standard error and assessed by using one way ANOVA analysis followed by a Tukey's test (95% confidence) for multiple comparisons (SPSS version 17). The survival analysis was analyzed with Breslow test. P value of < 0.05 is considered statistically significant.

## Results

### Modulation of tissue IFN-γ, IL-17 and TGF-β by EJHE and EJHE-WR

**Figure 1:**
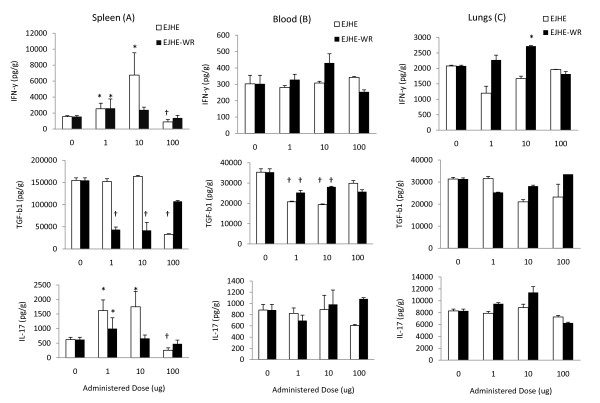
IFN-γ, TGF-β and IL-17 production levels in the spleen (A), blood (B) and lungs (C) of mice at 2 hours following administration of 0, 1, 10, and 100 μg EJHE or EJHE-WR (* indicates a significant increase or † indicates a significant decrease in comparison to 0 μg, see text for details).

To choose the best possible dose for the tissue cytokine-induced modulation over time by the extracts and other experiments, three different concentrations of EJHE and EJHE-WR and two-hour time point following administration were chosen [10]. Administration of EJHE (1-100 μg) significantly modulated and increased IFN-γ and IL-17 production in the spleen at 1 and 10 μg of EJHE (p < 0.0001 and p < 0.001 for IFN-γ and IL-17, respectively) (Figure 1A). At higher doses of EJHE (100 μg), however, IFN-γ and IL-17 levels were significantly less than the control values (p < 0.001). As for EJHE-WR, 1 μg/ml increased significantly IFN-γ and IL-17 production in the spleen (p < 0.05 and <0.001, respectively). This increase was not observed at higher doses. In addition, EJHE-WR at 1 and 10 μg, but not EJHE, suppressed significantly TGF-β level in the spleen (p < 0.005 and <0.001, respectively) (Figure 1A).

In blood, TGF-β levels were reduced after (1-100 μg) of EJHE and 1 and 10 μg of EJHE-WR administrations (p < 0.001 and <0.05, respectively), whereas blood IL-17 levels were not significantly modulated following either dose of EJHE or EJHE-WR (p > 0.05) (Figure 1B). In the lungs, 10 μg of EJHE-WR increased significantly IFN-γ level (p < 0.001) (Figure 1C).

### Modulation of tissue IFN-γ, IL-17 and TGF-β over 48 hours by EJHE and EJHE-WR

**Figure 2:**
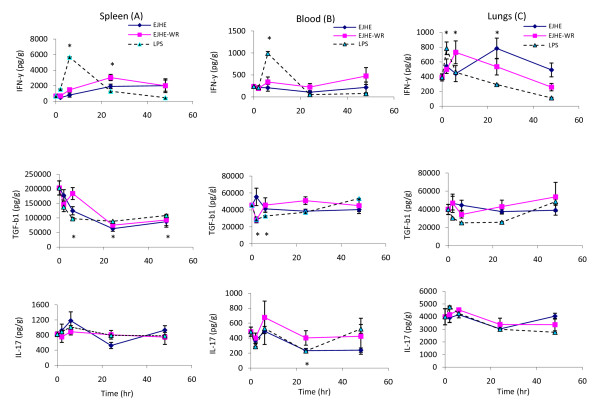
IFN-γ, TGF-β and IL-17 production levels in the spleen (A), blood (B) and lungs (C) of mice over 48 hours following administration of 10 μg EJHE, EJHE-WR or 2 μg LPS (*: indicates a significant change in comparison to 0 time, see text for details).

The tissue cytokines study indicated that 10 μg of either extract will be suitable dose for the kinetic study. A single i.p. injection of 10 μg EJHE or EJHE-WR into mice modulated significantly spleen cytokines level. EJHE or EJHE-WR at 10 μg increased significantly IFN-γ production in the spleen at 6-48 hours post i.p. administration (p < 0.01). The increase was highest 24 hours post administration of EJHE-WR and EHJE (Figure 2A). On the other hand, EJHE and EJHE-WR suppressed significantly spleen TGF-β1 production lasting 48 hours post administration (p < 0.001). The latter suppression was highest 24 hours post administration and stayed till 48 hours (Figure 2A). As for Il-17, EJHE (10 μg) exhibited a trend toward suppression of IL-17 level at 24 hours post administration (p > 0.05). This EJHE-induced suppression was not observed at 48 hours post administration. However, IL-17 levels did not change after i.p. administration of EJHE-WR (p > 0.05) (Figure 2A). Furthermore, i.p. administration of LPS increased significantly IFN-γ production over the first 6 hours of administration (p < 0.0001). This increase was accompanied with a significant reduction in TGF-β levels between 6-48 hours and with no change in IL-17 levels (Figure 2A).

EJHE at 10 μg did not induce any significant change in blood IFN-γ or TGF-β levels for 48 hours following i.p. administration (p > 0.05) but displayed a trend towards a decrease by 25-52% in blood IL-17 production 24-48 hours post i.p. administration (p > 0.05). Contrariwise, EJHE-WR displayed a trend towards an increase by 41-98% in blood IFN-γ levels between 6 to 48 hours post i.p. administration (p > 0.05) with a trend towards a decrease in TGF-β1 production 2 hours post administration (p > 0.05) and a trend towards an increase by 39% in blood IL-17 production 6 hours following i.p. administration (p > 0.05). LPS on the other hand, increased significantly IFN-γ production (311%) in blood 6 hours following i.p. injection. This maximum increase subsided below basal levels at 24 and 48 hours post administration (p < 0.0001) (Figure 2B). The latter modulation was accompanied with a significant suppression in blood TGF-β1 production over the first 6 hours post LPS administration (p < 0.001) and a significant reduction of blood IL-17 production at 24 h (p < 0.05) (Figure 2B).

A single i.p. injection of EJHE or EJHE-WR modulated the production of IFN-γ levels in mice lung tissues. EJHE-WR increased significantly IFN-γ production in the lungs over 24 hours post i.p. administration (p < 0.03). This increase was highest 6 hours post administration by 87% followed by a decline at 48 hours. Similarly, EJHE demonstrated a trend towards an increase in IFN-γ production for as long as 48 hours after administration with a maximum increase (101%) noticed 24 hours post i.p. injection (p > 0.05). On the other hand, neither EJHE nor EJHE-WR modulated IL-17 or TGF-β levels in the lungs. Furthermore, a single i.p. administration of LPS increased significantly (101%) IFN-γ production in the lungs 2 hours after administration followed by a decline over the next 6-48 hours after administration to lower levels than the basal IFN-γ levels (p < 0.002) (Figure 2C). Similarly, LPS did not induce any significant change in IL-17 levels in the lungs but demonstrated a trend towards a decrease of TGF-β levels over 2 to 24 hours post i.p. injection (Figure 2C).

### Modulation of IFN-γ, IL-17 and TGF-β1 in tumor-bearing mice

**Figure 3:**
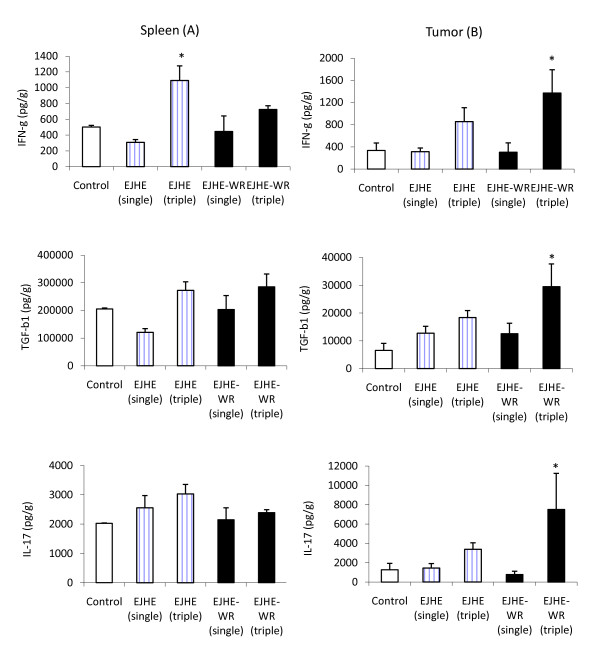
Effect of 10 μg EJHE and EJHE-WR on IFN-γ, TGF-β and IL-17 production levels in the spleen (A) and tumor (B) of Meth-A-bearing mice (*: indicates a significant change in comparison to the control, see text for details).

Twenty four hours post a single administration of EJHE or EJHE-WR (10 μg) did not modulate spleen or tumor IFN-γ, TGF-β and IL-17 production levels in tumor-bearing mice (Figure 3A &3B). However, when either extract was administered once daily for three consecutive days, spleen IFN-γ levels increased significantly by EJHE (p < 0.02) and showed a trend toward an increase by EJHE-WR (p >0.05) (Figure 3A). However, no changes in IL-17 or TGF-β1 levels were observed (p > 0.05) (Figure 3A). In addition, such administration modality increased significantly IFN-γ and IL-17 (p < 0.03) as well as TGF-β1 (p < 0.005) production by EJHE-WR within the tumor microenvironment of Meth-A-bearing mice (Figure 3B). Although a triple consecutive i.p. injections of EJHE showed trend toward increase in the production of the three cytokines within tumor microenvironment but without reaching statistical significance (Figure 3B).

### Survival of Meth-A tumor-bearing mice after treatment with EJHE and EJHE-WR

**Figure 4:**
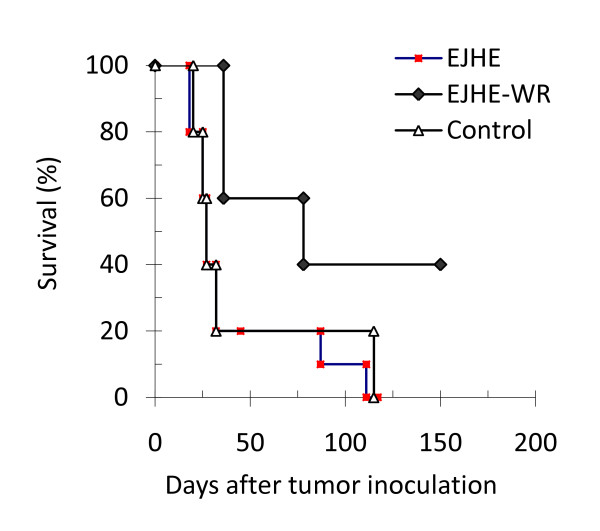
**Kaplan-Meier plot of mice inoculated i.p. with 2 × 10**^**6 **^**Meth-A cells followed by a three times a week injection of sterile PBS, EJHE (10 μg), or EJHE-WR (10 μg) for eight weeks**. Significant prolongation (*p < 0.007) of survival following administration of EJHE-WR-treated Meth-A-bearing mice was observed.

Kaplan-Meier plots of mice inoculated i.p. with 2 × 10^6 ^Meth-A cells followed by injections of sterile PBS, EJHE (10 μg), or EJHE-WR (10 μg) for eight weeks (as mentioned in the methods) are shown in Figure 4. The overall mortality patterns of the Meth-A-bearing mice shows that i.p. administration of EJHE-WR prolonged significantly (p < 0.007) the life-span of Meth-A-bearing mice with a median survival time of 78 days in comparison to 27 days for the control group. On the other hand, the mortality pattern of the EJHE treated group was found to overlap with the control group indicating that EJHE did not affect the survival of Meth-A-bearing mice (Figure 4).

## Discussion

Recently, it has been shown that water-extract of EJ, termed EJHE, provides an affluent source of polysaccharides and polar phenolic compounds such as procyanidins, flavonoid glycosides, and other molecules that may induce or modulate cytokines production providing distinguishable immunomodulatory and putative anticancer activity [4,10]. Thus, the fractionation of EJHE with n-butanol (EJHE-WR) was performed to concentrate polysaccharides, high molecular weight oligomeric procyanidins and related polyphenols and to exclude low molecular weight compounds recovered by n-butanol, in order to enhance cytokine modulatory activity [4].

Initially, three different concentrations of EJHE and EJHE-WR were chosen (1, 10, and 100 μg/ml) and their cytokine modulatory activities were sought in mice organs, including spleen and lungs following two hours of administration [10]. Collectively, this experiment demonstrated that the *in vivo *favorable cytokines modulatory effect in mice tissues as induction of IFN-γ and inhibition of TGF-β1 levels were best achieved by 10 μg of EJHE and EJHE-WR. Subsequently, the selected concentration was further utilized to investigate the tissue-specific effects of EJHE, EJHE-WR, in comparison with LPS on IFN-γ, IL-17, and TGF-β1 production levels over 48 hours and demonstrated that EJHE and EJHE-WR at 10 μg modulated and induced *in vivo *cytokines production in tissue- and time-specific manner. Administration of EJHE and EJHE-WR (10 μg) induced IFN-γ production in the spleen and lung tissues at 2-48 hours, and in blood at 6-48 hours after EJHE-WR administration only, whereas LPS induced maximum IFN-γ production in the spleen and blood at 6 hours, and at 2 hours in the lungs. Furthermore, the results illustrated that in contrast to the discerned induction of IFN-γ in the spleen, TGF-β1 was down-regulated in response to EJHE and EJHE-WR administration for as long as 48 hours. Likewise, blood, spleen, and lungs TGF-β1 levels were down-regulated upon LPS administration at 2-24, 2-48, and 2-24 hours, respectively.

Earlier studies *in vitro *showed that low concentrations of EJHE induced the production of proinflammatory cytokines (IL-12, IFN-γ, and TNF-α) whilst higher concentrations reduced the latter induction along with an increase in anti-inflammatory cytokine; IL-10 [10]. Similarly, water-extract of EJ leaves utilized at high concentrations inhibited LPS-induced proinflammatory cytokines; IL-6, IL-8, TNF-α; and IL-1β, IL-8, TNF-α from human mast cells and human lung epithelial cells, respectively, likely by inhibiting Nuclear factor kappa B (NF-κB) activation [16,17]. The latter coincides with the inhibition of proinflammatory cytokines and the increase in anti-inflammatory cytokine; IL-10, at higher EJHE concentrations [10]. Correspondingly, the data in the present study demonstrated a similar action *in vivo *as higher concentration (100 μg/ml) inhibited IFN-γ production in the spleen and lung tissues probably in a likewise manner, i.e. inhibition to NFκB, while on the contrary lower concentration (1-10 μg/ml) induced IFN-γ production and could be through increasing NF-κB activity [18].

Since EJHE and EJHE-WR induced and modulated cytokines in several organs, their cytokine modulatory mechanism might be postulated by the compounds they contain. These extracts contain mainly polysaccharides and polar phenolic compounds like procyanidins and flavonoid glycosides. Such compounds stimulate antigen-presenting cells (APCs); dendritic ells (DCs) and macrophages, via the expressed Toll-like Receptors (TLRs) [19]. The latter cells induce IL-12 levels through the activation of NF-κB [10], and APCs-derived IL-12 may preferentially stimulate T and B cells *in vivo *[20-23] (besides its action on NK cells) that in turn can influence the development of Th1 response and thus supports IFN-γ production [24,25]. The recognition of the polysaccharides with procyanidins and flavonoid glycosides by TLRs-expressed on DCs, macrophages, as well as B and T cell subtypes [22,26] induces subsequent engagement of signaling pathways that explain the timing of cytokine secretion pattern as apparent in IFN-γ highest induction at 2 or 6 hours upon LPS administration versus 24-48 hours after EJHE and EJHE-WR administration. Ostensibly, this differential induction time proposes that molecules within EJHE and EJHE-WR induce cytokine release in more complex pathways involving several intracellular signaling in comparison with LPS. Still, other non-immune cellular sources could be implicated in the cytokines responses depicted herein. Apart from immune cells, TLRs are expressed on tissues such as the lung epithelial cells [27,28]. Type 2 alveolar epithelial cells, for instance, have been shown to express mRNA for TLR2 and TLR4 and to have functional response with the release of cytokines in response to LPS [29].

Moreover, it cannot be ruled out that the changes observed in the present study may occur when EJHE-, EJHE-WR-, and LPS-induced IFN-γ bind to T cell populations in a paracrine fashion probably via employment of the JAK-STAT signaling pathway [30,31]. Generally, within a period of hours, upon the accumulation of activated STATs in the nucleus the signal decays and the STATs are re-exported back to the cytoplasm for a new cycle of signaling [32]. IFN-γ inhibits the TGF-β-induced phosphorylation of Smad3, the subsequent binding of Smad3 with Smad4, and the accumulation of Smad3 in the nucleus. Furthermore, upon IFN-γ signaling through Jak1 and STAT1 proteins and activation of NFκB, IFN-γ induces Smad7 expression which prevents the interaction of Smad3 with the TGF-β receptor leading to inhibition of TGF-β signaling and so reduction in its levels [33,34]. Probably, in this way, the crosstalk between the IFN-γ and TGF-β signals lead to the changes depicted herein.

Furthermore, Treg cells expressing Foxp3 are major source of TGF-β1 which regulates their generation as well [35,36]. Mice with a T cell-specific deletion of the *Tgfb1 *gene have enhanced Th1 cells proliferation, activation, and differentiation and TGF-β1, produced by Foxp3^+ ^Treg cells, was required to inhibit Th1 cells differentiation [36]. Therefore, TGF-β1 suppression upon EJHE, EJHE-WR, and LPS administration supported Th1 development and thus the enhanced IFN-γ production. Additionally, taking into account the delicate balance in the cytokines' network, the information conveyed by an individual cytokine depends on the pattern of regulators to which a cell is exposed, and not on one single cytokine [37]. Recent studies have established that TGF-β1 not only regulates the generation of Foxp3^+ ^Treg cells but also acts as an essential regulator of Th17 cell differentiation from naïve CD4^+ ^T cells together with IL-6 [35,38,39]. In the current study, the disappearance of a crucial cytokine for Th17 differentiation as TGF-β1 in the spleen upon EJHE, EJHE-WR, and LPS administration could foretell the absence of absolute Th17 cells and thus predict no change in IL-17 levels noticed herein.

Commonly, the immune system may be either compromised or suppressed in cancer patients. In view of the cytokine modulatory effects of EJHE and EJHE-WR, it was of considerable interest to investigate if administration of EJHE and/or EJHE-WR can restore the balance of proinflammatory/anti-inflammatory cytokines in the tumor microenvironment of Meth-A tumor-bearing mice. In the present study, Meth-A cells were injected into the s.c. layer which consists of loose connective tissue permitting the injected cells to diffuse easily [40]. The local microenvironment of Meth-A cells administered s.c. is complex since, besides fibroblasts, the microenvironment can also comprise numerous types of resident or infiltrating cells, like endothelial cells, muscular cells as well as granulocytes, macrophages, and lymphocytes [40,41]. The results from the present study demonstrated that a single i.p. administration of 10 μg EJHE and EJHE-WR did not modulate cytokines in the spleen of tumor-bearing mice as seen in healthy mice as well as in the tumor microenvironment. For instance, 24 hours post administration of EHJE or EJHE-WR, the TGF-β to IFN-γ ratio in the spleen of healthy mice became ~30 in comparison to 300 in PBS-injected mice whereas this ratio either did not change or increased upon administration of EHJE and EHJE-WR in tumor-bearing mice. This is due to the systemic immunosuppressive behavior of the tumor and within its environment which enhances the depolarization of macrophages, DC and neutrophils, recruitment of Treg cells within tumor microenvironment, and lodging of the latter cells in lymphoid organs resulting in producing suppressive cytokine such as TGF-β [42]. Because a single injection did not cause substantial change in cytokine modulation in tumor-bearing mice, triple injections once every 24 hours for three consecutive days were thought to induce a significant cytokine modulation. The latter regimen by EJHE increased spleen IFN-γ production whereas EJHE-WR increased significantly IFN-γ, and unexpectedly TGF-β1 and IL-17 production levels within the tumor microenvironment of Meth-A-bearing mice.

The induction of IFN-γ in the tumor microenvironment and spleen of Meth-A-bearing mice by triple i.p. injection of EJHE-WR and EJHE, respectively, is probably connected with their constituents enhancing effect allowing the intratumoral penetration of antitumor effectors and polarizing towards Th1 response from macrophages and DCs produced IL-12 which in turn can support IFN-γ production noticed within the microenvironment [43]. However, this favorable induction of IFN-γ by EJHE-WR within the tumor microenvironment was accompanied by TGF-β1 induction, probably along with an inflammatory cytokine e.g. TNF-α [10,42] that is common in such microenvironment, may further supports Th17 differentiation and thus induce IL-17 production as well [44]. This could be explained by the fact that tumor-bearing mice have a systemic increase in their depolarized immune cells (DC, macrophages, neutrophils) and Treg that would secrete more TGF-β upon stimulation and have less polarized cells that enhance DC-Th1 pathway to produce IFN-γ [42,45]. Furthermore, repeated administration of EJHE or EJHE-WR may resulted in an increase in tumor-infiltrating CD4+CD25+Foxp3+ Treg cells that led to increase in TGF-β in tumor microenvironment [42]. The latter note can also explain the observed results in the spleen of tumor-bearing mice. It is worth to investigate the various cellular populations that were recruited to or modulated in the tumor microenvironment and spleen of Meth-A-bearing mice upon repeated i.p. administration of EJHE and EJHE-WR.

The present results also demonstrated a prolonged survival of mice inoculated i.p. with Meth-A cells throughout long period i.p. administration of EJHE-WR in comparison with Meth-A-bearing mice treated with EJHE (10 μg) and PBS. Previous work showed that a fractionated water-extract of EJ leaves with butanol recovered high molecular weight procyanidin oligomer which displayed the highest cytotoxic activity *in vitro *against two human oral tumor cell lines [4]. Particularly, fractionation of EJHE with n-butanol to obtain EJHE-WR provided an abundant source of high molecular weight procyanidin oligomer together with polysaccharides and polar phenolics compounds possessing low anti-inflammatory activity, meanwhile, exerting immunostimulating potentials [10] and can also be involved in antitumor and cytotoxic activities [4]. In addition, repeated EJHE-WR administration produced higher IFN-γ in the tumor microenvironment which should enhance T cytotoxic and NK cell activities, as well as increased endogenous IL-17 which was found to reduce tumor growth [46]. Previous studies displayed critical functions for IFN-γ dependent tumor rejection of transplanted tumors and MCA-mediated tumorigenesis in mice [47,48]. The immune response against Meth-A tumor cells appeared to be closely associated with augmented IFN-γ production and CTL activity against tumor which can injure tumor-feeding vessels in tumor tissue [3,41]. However, further studies are needed to clarify the antitumor activities of EJHE-WR constituents involved in Meth-A bearing mice.

## Conclusions

EJHE and EJHE-WR cytokine modulatory potentials have been characterized in tissues from normal and tumor-bearing mice. It is clear that multidimensional factors partake in depicting the responses noticed herein. In that respect, (i) the nature of the immunomodulating agent that elicits the immune response in each occasion, (ii) the type and timing of cytokine secretion either by the stimulating APCs, T and B lymphocytes through TLRs mediated pathways, or other onlooker cell populations, and (iii) the impact of tissue/organ/tumor being studied with its burden and phenotype of residing cell populations that also cannot be excluded. Furthermore, immunomodulators which can be used for long period with minimum side effects are substantial in the cancer therapy. Ordinarily, targeted cancer monotherapy can end up with bypass mechanisms which in turn forced the employment of either combination therapy or agents that interfere with multiple cell-signaling pathways as a current paradigm for most treatments.

## Competing interests

The authors declare that they have no competing interests.

## Authors' contributions

HA carried out the experimental work, statistical analysis and drafted the manuscript. NQ participated in the designs of the animal experiments and helped in the animal work. FQ designed and supervised the extraction procedures. MB participated in drafting some of the experiments and revised critically the manuscript. KM conceived of the study, and participated in the tumor development, immunoassays, design of experiments, statistical analysis and helped in drafting the manuscript. All authors read and approved the final version of the manuscript.

## Pre-publication history

The pre-publication history for this paper can be accessed here:

http://www.biomedcentral.com/1472-6882/11/9/prepub
